# TRIM25 regulates oxaliplatin resistance in colorectal cancer by promoting EZH2 stability

**DOI:** 10.1038/s41419-021-03734-4

**Published:** 2021-05-08

**Authors:** Sha Zhou, Jianhong Peng, Liuniu Xiao, Caixia Zhou, Yujing Fang, Qingjian Ou, Jiayi Qin, Mengzhong Liu, Zhizhong Pan, Zhenlin Hou

**Affiliations:** 1 Department of Radiation Oncology, Sun Yat-Sen University Cancer Center, State Key Laboratory of Oncology in South China; Collaborative Innovation Center for Cancer Medicine, Guangzhou, 510060 China; 2 Department of Colorectal Surgery, Sun Yat-Sen University Cancer Center, State Key Laboratory of Oncology in South China; Collaborative Innovation Center for Cancer Medicine, Guangzhou, 510060 China; 3 Department of Intensive Care Unit of Tongji Hospital, Huazhong University of Science and Technology, Wuhan, 430030 China

**Keywords:** Cancer therapeutic resistance, Oncogenes

## Abstract

Resistance to chemotherapy remains the major cause of treatment failure in patients with colorectal cancer (CRC). Here, we identified TRIM25 as an epigenetic regulator of oxaliplatin (OXA) resistance in CRC. The level of TRIM25 in OXA-resistant patients who experienced recurrence during the follow-up period was significantly higher than in those who had no recurrence. Patients with high expression of TRIM25 had a significantly higher recurrence rate and worse disease-free survival than those with low TRIM25 expression. Downregulation of TRIM25 dramatically inhibited, while overexpression of TRIM25 increased, CRC cell survival after OXA treatment. In addition, TRIM25 promoted the stem cell properties of CRC cells both in vitro and in vivo. Importantly, we demonstrated that TRIM25 inhibited the binding of E3 ubiquitin ligase TRAF6 to EZH2, thus stabilizing and upregulating EZH2, and promoting OXA resistance. Our study contributes to a better understanding of OXA resistance and indicates that inhibitors against TRIM25 might be an excellent strategy for CRC management in clinical practice.

## Introduction

Colorectal cancer (CRC) is the third most common cancer worldwide and the fourth leading cause of cancer-related mortality^1^. Surgical resection plus oxaliplatin (OXA)-based chemotherapy is the most frequently used therapeutic strategy for patients with CRC^2^. However, cancer cells eventually develop chemoresistance, which is considered the major cause of treatment failure in patients with metastatic cancer and is a major limitation of the efficacy of chemotherapy drugs in clinical practice^3^. Thus, revealing the underlying mechanism and discovering new therapeutic targets are necessary to improve treatment outcomes for patients with CRC.

Cancer stem cells (CSCs) are a special cellular sub-population that exhibit self-renewing and tumorigenic capacities^4,5^. In recent years, emerging evidence has suggested that the presence of CSCs is a key factor in tumor resistance to chemotherapeutic drugs^6^. The expression of stem cell markers, such as CD133 or CD44, is associated with chemoresistance^7,8^. In glioblastoma, CD133-positive CSCs have demonstrated resistance to chemotherapy, by increasing the expression of the drug resistance gene BCRP1, the DNA mismatch repair gene MGTMT, and some antiapoptotic genes^7^. In hepatocellular carcinoma (HCC), upregulation of CD44 has been proven to be related to resistance to anticancer drugs, including 5-fluorouracil, cisplatin, and irinotecan^9^. Therefore, triggering CSCs to exit the stem-like state, which would result in increased response of cancer cells to chemotherapy, is a novel means of overcoming chemotherapy resistance.

Epigenetic regulation of transcriptional programs, including DNA methylation, non-coding RNAs, and histone posttranslational modifications (e.g., acetylation and methylation), is a key driver of the self-renewal capacity^10–12^. Enhancer of zeste 2 polycomb repressive complex 2 subunit (EZH2) is a critical component of the epigenetic polycomb repressive complex 2 (PRC2) and silences target genes via tri-methylating histone H3 on lysine 27 (H3K27me3)^13^. Upregulation of the H3K27me3 methyl-transferase EZH2 has been observed in various cancers. EZH2 is associated with a high proliferation rate, aggressive tumor subtypes, and poor outcome of patients with cancer^14^. In addition, EZH2 is reported to contribute to chemotherapy response, and EZH2 inhibitors have been proven to reverse drug resistance in cancers^2,15^. Recent studies showed that EZH2 has an essential role in maintaining CSC properties in multiple cancer types, including breast cancer, prostate cancer, and glioblastoma^16–18^. In the context of CRC, EZH2 has been proven to contribute to the CSC state by modulating key pathways such as Wnt/β-catenin and Hedgehog signaling, where high EZH2 activity has been shown to designate the CSC population^15^. Therefore, EZH2 might be a promising target for cancer therapy.

Tripartite motif containing 25 (TRIM25) is a member of the tripartite motif (TRIM) family that functions in multiple RNA-dependent pathways^19^. Like a typical TRIM protein, TRIM25 consists of an N-terminal tripartite motif, or RBCC motif, and a C-terminal SPRY domain^20–22^. Accumulating evidence suggests that TRIM25 has a key role in many physiological disorders, predominantly by regulating ubiquitination of its target protein. For example, TRIM25 is crucially involved in interferon signaling by mediating K63-linked polyubiquitination of RIG-1, which is important for host antiviral innate immunity^21^. Moreover, TRIM25 has recently been reported to be essential for tumorigenesis^23–26^. High expression of TRIM25 has been demonstrated in a variety of cancers, such as CRC, lung cancer, and breast cancer^23–25^. In HCC, TRIM25 promotes cancer cell survival and growth through targeting Keap1-Nrf2 pathway^26^. Despite extensive research on TRIM25 in cancer, the role of TRIM25 in regulating drug resistance remains largely unknown.

In the present study, we revealed a previously unknown mechanism of OXA chemoresistance. We demonstrated that EZH2 is regulated by TRIM25 in CRC cells. TRIM25 inhibits the binding of TRAF6, an E3 ubiquitin ligase, to EZH2, which stabilizes EZH2 to promote OXA resistance. Our findings suggest that TRIM25 is a novel epigenetic regulator, and targeting the TRIM25–EZH2 pathway might be a promising approach to CRC treatment.

## Materials and methods

### Cell lines and cell culture

The CRC cell lines (SW48 and SW480) were purchased from the American Type Culture Collection (ATCC, Manassas, VA, USA) and cultured under conditions as recommended. All cell lines were authenticated using short tandem repeat (STR) fingerprinting and negatively tested for mycoplasma contamination before experiments.

### Patients and tissue specimens

The formalin-fixed, paraffin-embedded CRC tissues (*n* = 223) were obtained between November 2007 and December 2012 at the Sun Yat-sen University Cancer Center. The patient characteristics are summarized in Supplementary Table 1. All patients were treated with a FOLFOX or XELOX regimen and followed up with regular surveillance at our hospital. Approvals from the ethical committee of Sun Yat-sen University Cancer Center and prior patient’s consents were previously obtained for the use of these clinical specimens for research purpose.

### Western blotting analysis

Protein concentrations were determined using the BCA Protein Assay Kit (Beyotime, P0012). Primary antibodies used in this study are anti-TRIM25 (Proteintech, 12573-1-AP), anti-cleaved PARP (CST, #5625), anti-cleaved Caspase 3 (CST, #9664), anti-EZH2 (CST, #5246), anti-TRAF6 (CST, #8028), anti-Flag tag (Proteintech, 60002-1-Ig), anti-HA tag (Proteintech, 51064-2-AP), anti-His tag (Proteintech, 10001-0-AP), and anti-GAPDH (CST, #5174).

### Immunohistochemistry

Immunohistochemistry (IHC) analysis using paraffin-embedded CRC specimens was conducted following standard manufacturer’s protocols as described previously. Primary antibodies anti-TRIM25 (Proteintech, 67314-1-Ig) and anti-EZH2 (Cell Signaling Technology, #5246) were used for IHC staining. IHC staining was evaluated by two independent gastrointestinal pathologists blinded to the patients’ characteristics and clinical outcomes. Final IHC score was calculated based on both the extent and the intensity of staining. The staining extent that scored according to the percentage of positively stained cells ranged from 0 to 3 (0, 0–25%; 1, 25–50%; 2, 50–75%; and 3, 75–100%), although the intensity of staining was scored as 0 (negative staining), 1 (weak staining), 2 (moderate staining), and 3 (strong staining). The Cutoff Finder program was used to determine the optimal cutoff for TRIM25 and EZH2 expression. Specimens with the final scores ≥ 4 were defined as high expression, and specimens with the final scores <4 were defined as low expression.

### RNA interference

For knockdown of TRIM25, negative control or target gene shRNAs were co-transfected into HEK293T with pHelper and pEnv. The virus was harvested from the transfected HEK293T cells, and the target cells were then infected with the viral supernatants for 2 consecutive days. Stable CRC cell lines were selected by treating with puromycin (2 μg/mL) for 10 days. Target sequences for shRNAs are shown in Supplementary Table 3.

### Cell viability assay

CRC cells (5 × 10^3^ cells/well) were seeded in 96-well plates and treated with corresponding processes. At the indicated time point, CCK8 was added into the wells and incubated with cells according to the product manual. Then the Thermomax microplate reader was used to measure the absorbance of each well at wavelength of 450 nm (A450).

### Apoptosis assay

For cell apoptosis assay, CRC cells treated with oxaplatin were harvested and stained with annexin V-FITC and propidiumiodide (PI) using an annexin V-FITC/PI staining kit (BD Pharmingen™, San Diego, CA, USA). After incubation at room temperature for 15 min, the cells were analyzed by flow cytometry.

### Sphere formation assay

CRC cells were first collected and fully resuspended in the medium for sphere formation. Next, the density of the cell suspensions was counted using an electron microscope. Then, CRC cells (1000 cells/well) were seeded in six-well ultra-low attachment plates and cultured with the medium for sphere formation. Tumor spheres were measured every other day.

### Immunoprecipitation (IP) and ubiquitination analysis

Cancer cells were washed with cold phosphate-buffered saline (PBS) and lysed in NP-40 lysis buffer at 4°C. For EZH2 ubiquitination detection, 24 hours after transfection, cells were treated with the proteasome inhibitor MG132 (10 µM) for 6 h and lysed with NP-40 lysis buffer supplemented with protease-inhibitor cocktail. Total cell lysates were incubated with the appropriate primary antibodies overnight and subsequently rotated with protein A/G beads for 2~4 h at 4°C. The beads were then washed with NP-40 lysis buffer for three times, mixed with 2× sodium dodecyl sulfate sample buffer and boiled for 10 min. The co-precipitates were analyzed by immunoblotting analysis using a chemiluminescence method.

### Xenograft tumor model

Male BABL/c nude mice (4–5 weeks old) were randomly divided into four groups and used to evaluate the clinical benefits of targeting TRIM25. For limiting dilution assay, indicated number (1 × 10^6^, 1 × 10^5^, 1 × 10^4^) of SW480 cells transfected with/without TRIM25 or shTRIM25 was subcutaneously injected to the left flank of each nude mouse (eight mice/group). Tumor volumes were then measured every four days and the tumor initiating cell frequency was calculated by ELDA software. For tumors treated with OXA, TRIM25-overexpressing SW480 cells (5 × 10^5^) treated with or without EZH2 inhibition were suspended in 100 μl PBS and subcutaneously injected to the left flank of each nude mouse (6 mice/group). One week later, the mice were intraperitoneally injected with OXA (5 mg/kg, twice a week). Tumor growth curve was measured for 3 weeks and the volume of tumor was calculated as length × Width^2^ × 1/2. At the end of the study, we surgically removed the tumors from the sacrificed mice. The animal experiments were conducted according to the Animal Study Guidelines of the Ethics Committee of Sun Yat-Sen University.

### Statistical analysis

Statistical analyses were performed using SPSS version 24.0 software. All data were presented as the mean ±SD. Statistical tests used in this study included the two-tailed Student’s *t* test, *χ*^2^ test and log-rank test. *P* value < 0.05 was considered statistically significant.

## Results

### High expression of TRIM25 predicts recurrence in patients with CRC

**Fig. 1:**
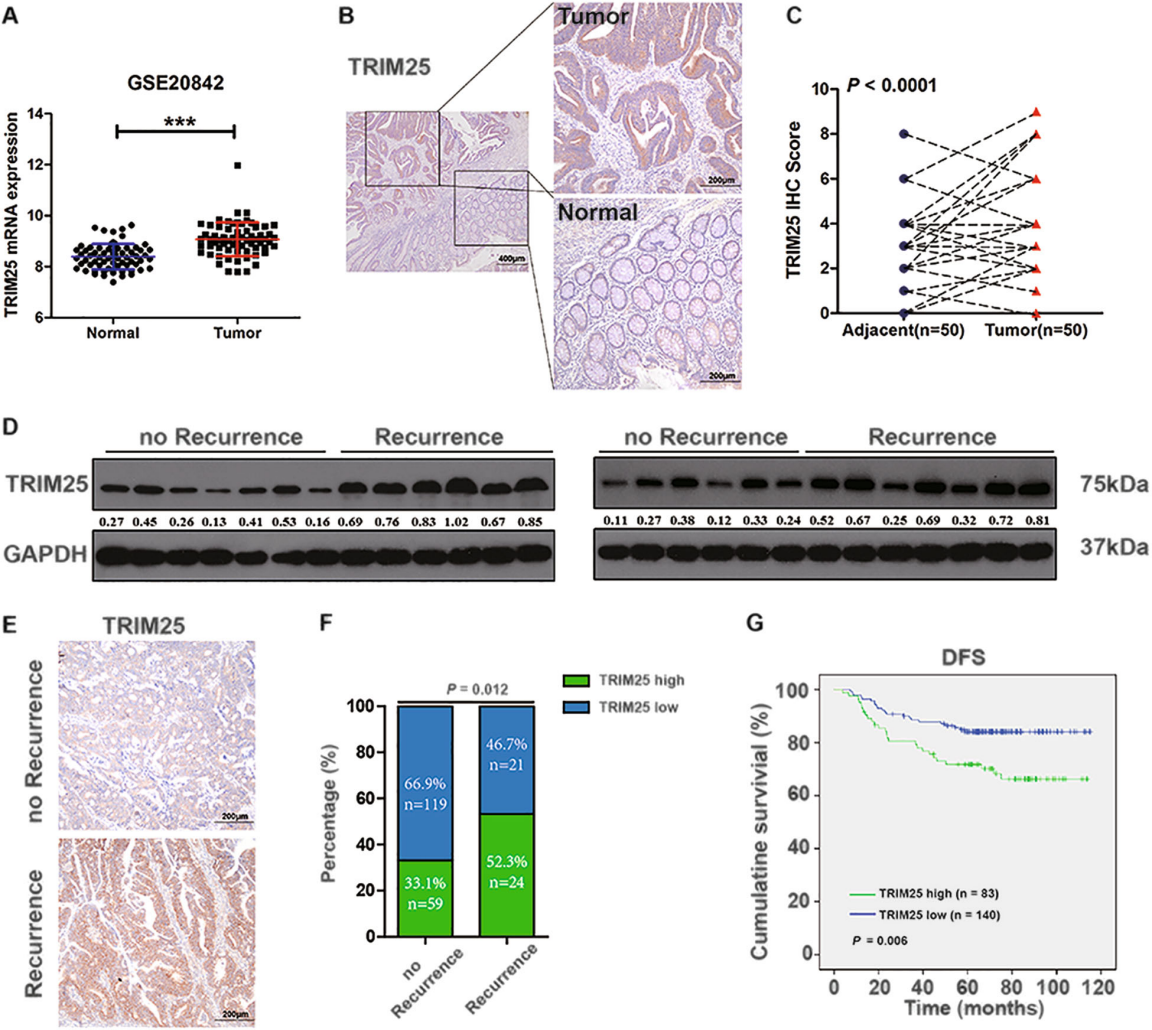
High expression of TRIM25 is associated with recurrence in colorectal cancer. **A** Analysis of TRIM25 expression in CRC and adjacent normal tissues from publicly available GEO data set (GSE20842), as assessed using a *t* test. **B** Representative IHC images of TRIM25 in colorectal tumor or normal tissues. Scale bar, 20 μm. **c** IHC scores of TRIM25 levels in paired normal and CRC samples are shown as a symbol-line plot. Expression data for normal and tumor tissue from a given individual are linked with dashed lines. Differences were assessed using a paired two-tailed *t* test. **D** Western blotting analysis showing the level of TRIM25 in 26 primary tumor tissues from patients with stage III CRC who were treated with OXA-based chemotherapy, including 13 samples from patients who developed recurrence and 13 samples from patients who did not develop recurrence after surgical resection. **E** Representative IHC images of TRIM25 in 223 CRC tissues with or without recurrence. Scale bar, 200 μm. **F** Relationship between TRIM25 expression and CRC recurrence, as assessed using a *χ*^2^ test. **G** Comparison of disease-free survival between patients with high or low TRIM25 expression, as assessed using a log-rank test.

We analyzed publicly available CRC mRNA expression profiles (GSE20842) obtained from the NCBI and found that the mRNA expression of TRIM25 is elevated in CRC tissues compared with that in normal tissues (Fig. 1A). To confirm our in silico observations, we detected TRIM25 protein levels in CRC samples. IHC staining showed that TRIM25 is present in both the cytoplasmic and nuclear regions of CRC cells. In the paired samples, 66.0% (33/50) of the CRC tissues had a higher TRIM25 IHC score and 24.0% (12/50) of the CRC tissues had a lower TRIM25 IHC score than the adjacent normal tissues, and the remaining 10.0% (5/50) of the CRC tissues and adjacent normal tissues had the equal TRIM25 IHC score (Fig. 1B, C). To explore the potential role of TRIM25 in CRC therapy, we initially evaluated the TRIM25 level in 26 primary tumor tissues from patients with stage III CRC that were treated with OXA-based chemotherapy. The results showed that the TRIM25 level in OXA-resistant patients who developed recurrence during the follow-up period was significantly higher than that of patients who had no recurrence (Fig. 1D and Supplementary Fig. S1a). We further analyzed TRIM25 expression in 223 paraffin-embedded human CRC specimens from patients who received Xelox or FOLFOX treatment after surgery (the patient characteristics are summarized in Supplementary Table 1). Representative IHC staining confirmed that TRIM25 levels were markedly increased in patients with CRC with tumor relapse (Fig. 1E). Patients with high TRIM25 levels demonstrated an observably higher recurrence rate than those with low TRIM25 levels (28.9% vs 15.0%, *P* = 0.012, Fig. 1F and Table 1). Furthermore, univariate analysis revealed that advanced age and high TRIM25 expression were associated significantly with worse OS and DFS (*P* = 0.006, Fig. 1G and Supplementary Fig. S1b). On multivariate analysis, TRIM25 expression was an independent prognostic factor for DFS (Supplementary Table 2). Collectively, the above observations suggest that elevated TRIM25 levels contribute to the progression of CRC and are associated with the failure of OXA-based chemotherapy.

**Table 1:** Association of TRIM25 expression with patient characteristics.

Characteristic	TRIM25 expression		*P* value
	Low	High	
*Age (years)*			0.245
<56	72 (51.4%)	36 (43.4%)	
≥56	68 (48.6%)	47 (56.6%)	
*Sex*			0.332
Male	75 (53.6%)	50 (60.2%)	
Female	65 (46.4%)	33 (39.8%)	
*Tumor location*			0.914
Right-sided	55 (39.3%)	32 (38.6%)	
Left-sided	85 (60.7%)	51 (61.4%)	
*Primary tumor size*			0.668
<4.5 cm	75 (53.6%)	42 (50.6%)	
≥4.5 cm	65 (46.4%)	41 (49.4%)	
*T stage*			0.012
T1-2	66 (47.1%)	25 (30.1%)	
T3-4	74 (52.9%)	58 (69.9%)	
*N stage*			0.522
N1	92 (65.7%)	58 (69.9%)	
N2	48 (34.3%)	25 (30.1%)	
*TNM stage*			0.059
T1–3N1M0	47 (33.6%)	18 (21.7%)	
T4NanyM0 or TanyN2M0	93 (66.4%)	65 (78.3%)	
*Recurrence*			0.012
Yes	21 (15.0%)	24 (28.9%)	
No	119 (85.0%)	59 (71.1%)	

### TRIM25 confers OXA resistance in CRC cells in vitro

**Fig. 2:**
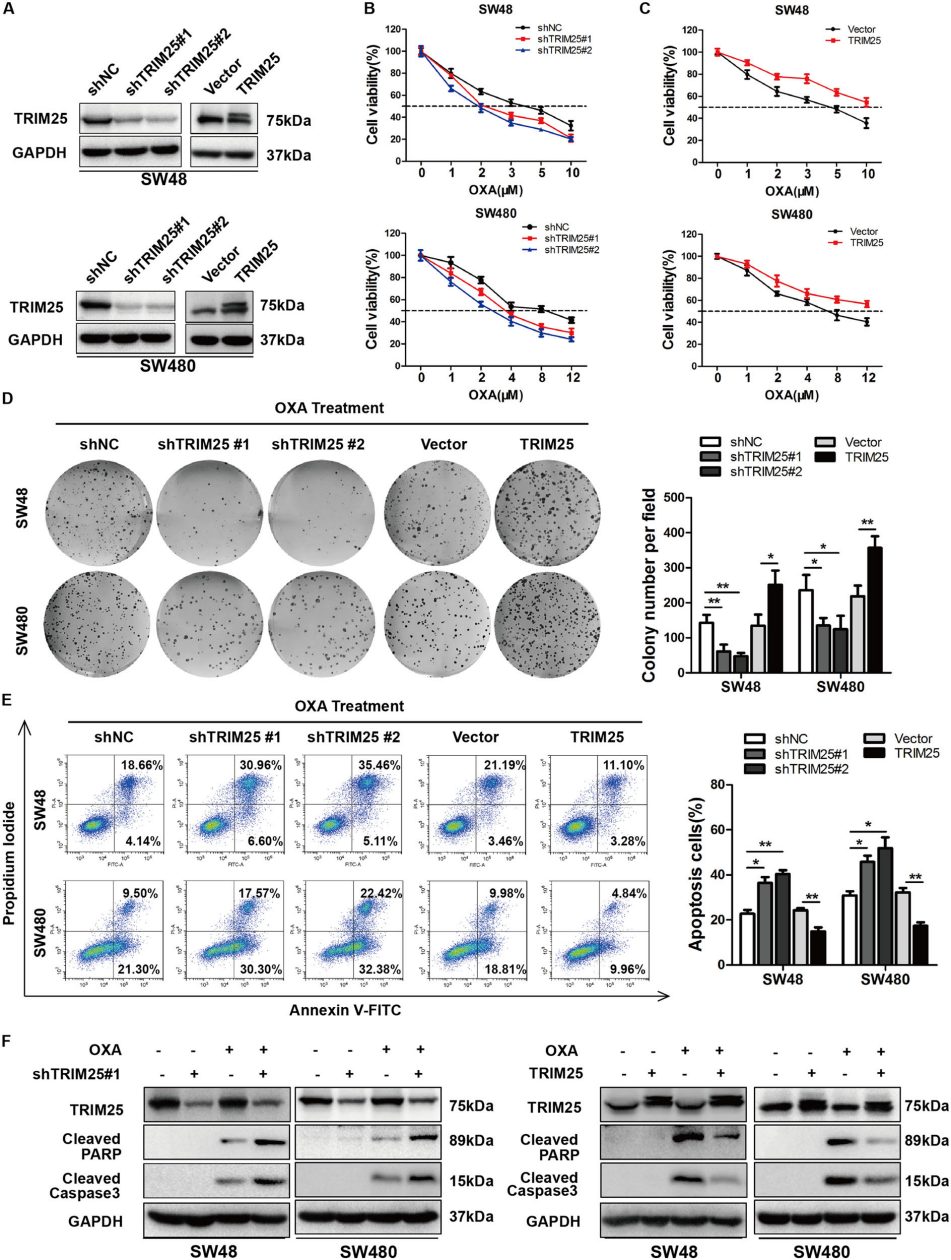
TRIM25 influences the sensitivity of CRC cells to oxaliplatin. **A** Western blotting analysis of TRIM25 in SW48 and SW480 cells transfected with shTRIM25 or a TRIM25-expressing plasmid. GAPDH was used as the loading control. **B**–**C** A CCK8 assay was used to measure the viability of the indicated cells treated with different concentrations of OXA for 48 h. **D** Colony-formation ability (left panel: representative images; right panel: quantification of colony numbers) of the indicated cells treated with OXA (2 μM). **E** Annexin V-FITC and PI staining showing apoptosis in the indicated CRC cells treated with OXA (30 μM) for 48 h. Left panel: representative images; right panel: Quantification of apoptotic cells. **F** Western blotting analysis of cleaved caspase 3 and cleaved PARP in the indicated cells treated with or without OXA (30 μM) for 48 h. Data are represented as the mean ±SD of three independent experiments. *P < 0.05, **P < 0.01, **P < 0.001.

We then examined whether TRIM25 was associated with resistance to OXA-based therapy in preclinical models. First, we established stable TRIM25-knockdown and overexpressing cells from the SW48 and SW480 CRC cell lines (Fig. 2A). A CCK8 assay showed that the IC50 values for OXA were decreased in the TRIM25-knockdown cells and increased in the TRIM25-overexpressing cells (Fig. 2B, C). Consistently, in the presence of OXA, TRIM25-knockdown dramatically inhibited, whereas TRIM25 overexpression enhanced, the colony-formation ability of SW48 and SW480 cells (Fig. 2D). Moreover, compared with the control cells, knockdown of TRIM25 resulted in significantly increased OXA-induced apoptosis of CRC cells, whereas overexpression of TRIM25 reduced OXA-induced apoptosis (Fig. 2E). Measurement of cleaved caspase 3 and cleaved PARP further confirmed that knockdown of TRIM25 increased OXA sensitivity and upregulation of TRIM25 conferred OXA resistance (Fig. 2F). Taken together, these results revealed that TRIM25 confers OXA resistance in CRC cells.

### TRIM25 promotes stem cell properties of CRC cells

**Fig. 3:**
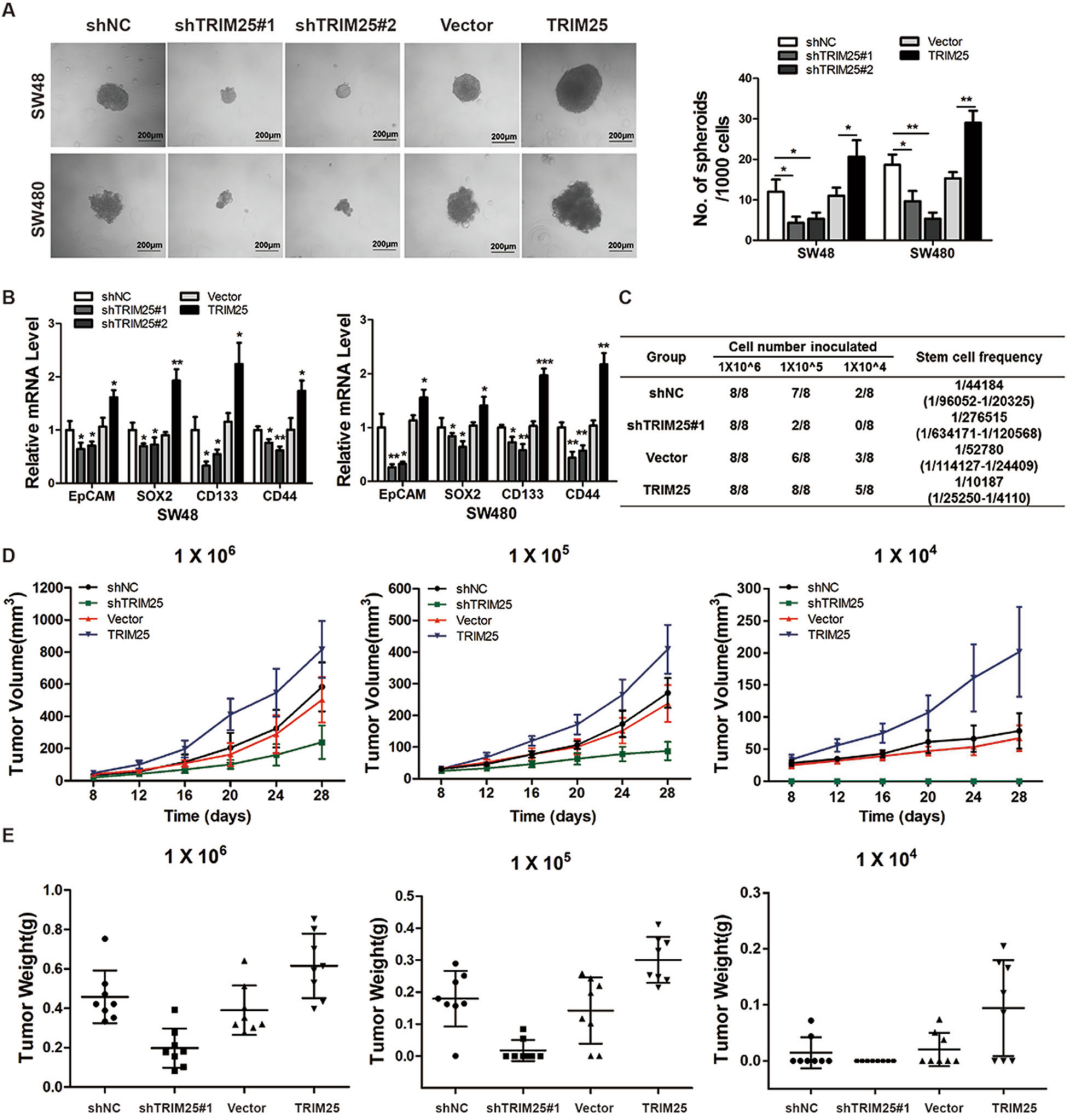
TRIM25 promotes stem cell properties of CRC cells. **A** Representative images (left panel) and quantification (right panel) of the in vitro sphere formation assay of TRIM25-knockdown and TRIM25-overexpressing SW48 and SW480 cells. Scale bar, 200 μm. Data are represented as the mean ±SD of three independent experiments. **B** Relative mRNA expression of EpCAM, SOX2, CD133, and CD44 in TRIM25-knockdown or TRIM25-overexpressing SW48 and SW480 cells. Data are represented as the mean ±SD of three independent experiments. **C** Limiting dilution data showing the stem cell frequency of the indicated group of SW480 cells from a tumorigenic mouse model. **D** Growth curves of each group of tumors. Indicated numbers of SW480 cells were inoculated into the nude mice and tumor volumes were measured every 4 days. Data are represented as the mean ±SD. **E** Each group of tumors was removed and weighted. Data represent the mean ±SD. *P < 0.05, **P < 0.01, ***P < 0.001 for indicated comparison.

As reported previously, stemness is believed to be responsible for chemotherapy resistance, thus we hypothesized that TRIM25 is involved in regulating CRC stemness and performed experiments to test our hypothesis. We conducted the sphere formation assay, and found a decrease in sphere numbers and sizes in TRIM25-knockdown cells compared with the corresponding control cells, whereas overexpression of TRIM25 enhanced the sphere formation ability of CRC cells (Fig. 3A). In addition, the expression of stem cell-related molecules, such as EpCAM, SOX2, CD133, and CD44, in SW48 and SW480 cells was markedly reduced after TRIM25 inhibition, whereas their expression increased after TRIM25 overexpression (Fig. 3B and Supplementary Fig. S2). Furthermore, limiting dilution analysis in vivo confirmed the markedly reduced stem cell frequency in TRIM25-knockdown SW480 cells (Fig. 3C), with the formation of smaller and lighter tumors than those formed by the control SW480 cells (Fig. 3D, E). In addition, no visible tumors could be formed in nude mice when 1 × 10^4^ TRIM25-knockdown SW480 cells were inoculated. These findings indicate the crucial role of TRIM25 in promoting the stem cell properties of CRC cells.

### TRIM25 regulates EZH2 stability in CRC cells

**Fig. 4:**
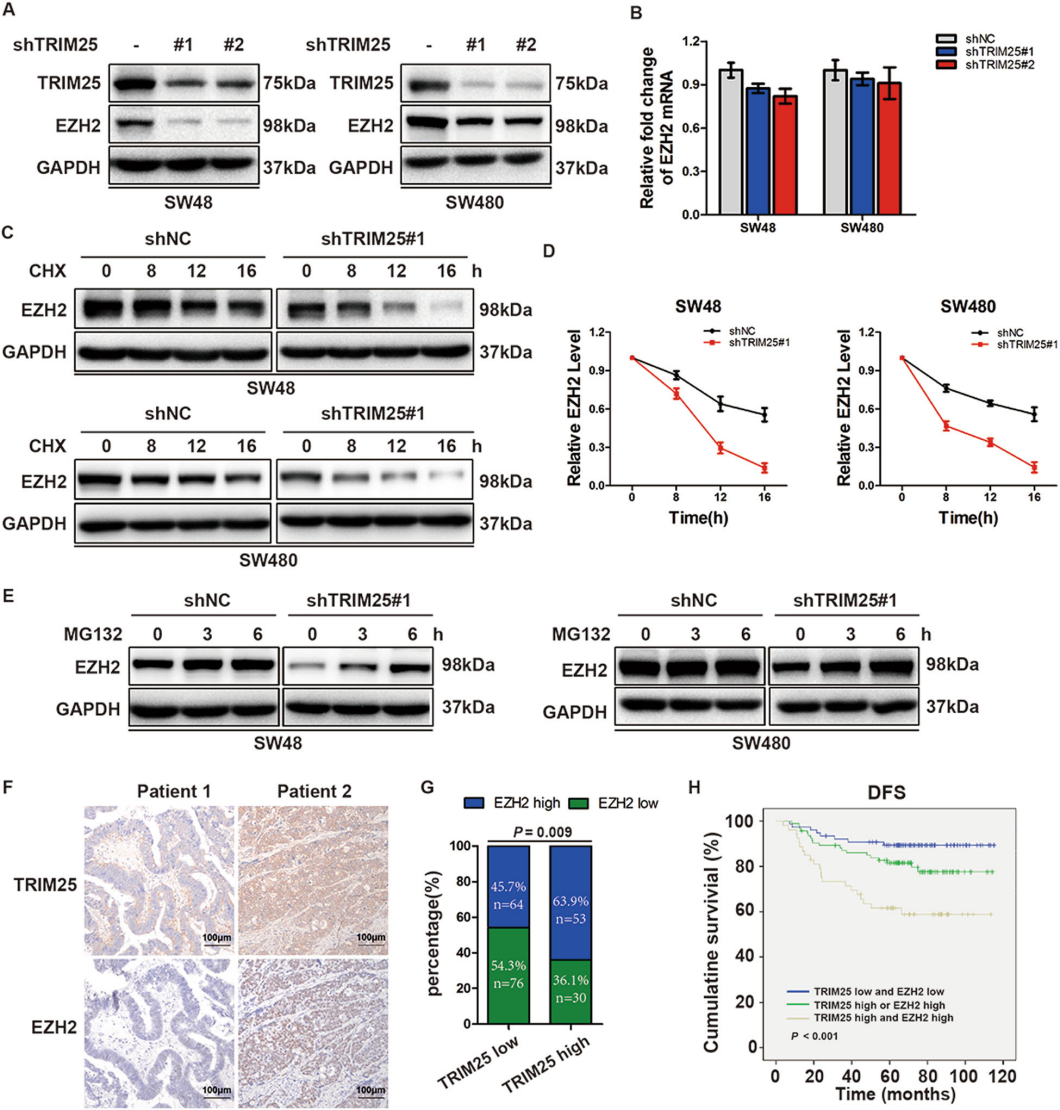
TRIM25 regulates EZH2 stability in CRC cells. **A** Western blotting analysis of EZH2 in control and TRIM25-knockdown SW48 and SW480 cells. **B** RT-PCR analysis of EZH2 mRNA in control and TRIM25-knockdown SW48 and SW480 cells. **C** EZH2 protein levels in control and TRIM25-knockdown SW48 or SW480 cells were detected at the indicated time points after CHX (20 μg/mL) treatment. **D** The relative level of EZH2 at each time point was normalized by the level of GAPDH. **E** Western blotting showing the effect of MG132 (10 μM) on EZH2 levels in control and TRIM25-knockdown SW48 or SW480 cells. **F** Representative IHC images of TRIM25 and EZH2 in two cases of CRC. Scale bar, 100 μm. **G** Relationship between TRIM25 expression and EZH2 expression in the same cohort of CRC samples, as assessed using the *χ*^2^ test. **H** Comparison of disease-free survival between patients with different TRIM25 and EZH2 expression patterns, as assessed using the log-rank test.

As the catalytic subunit of PRC2, EZH2 has an essential role in tumor progression. Previous studies revealed that targeting EZH2 inhibits CSC self-renewal and enhances the sensitivity of CRC to OXA (refs. ^2,15^). In the present study, we found that knockdown of TRIM25 decreased the protein level of EZH2 (Fig. 4A). However, there was no significant effect on EZH2 mRNA levels in both SW48 and SW480 cells (Fig. 4B), suggesting that TRIM25 might affect the stability of EZH2. To substantiate this assumption, we treated TRIM25-knockdown or control CRC cells with the protein synthesis inhibitor cycloheximide (CHX) and the proteasome inhibitor MG132. The results showed that knockdown of TRIM25 shortened the half-life of endogenous EZH2 protein in CRC cells after CHX treatment (Fig. 4C, D). The level of EZH2 was modestly increased in TRIM25-knockdown SW48 cells treated with MG132, and the same results were obtained in SW480 cells (Fig. 4E), implying that the ubiquitin–proteasome pathway might be involved in TRIM25-mediated stability of EZH2. Finally, we detected the EZH2 levels in the same cohort of CRC samples used for TRIM25 analysis using IHC staining and found that high EZH2 levels correlated significantly and positively with high TRIM25 levels (Fig. 4F). CRC samples with high TRIM25 levels showed a higher proportion of high EZH2 levels, whereas samples with low TRIM25 levels exhibited a lower proportion of high EZH2 levels (63.9% vs 45.7%, *P* = 0.009, Fig. 4G). Patients with CRC with high levels of TRIM25 and EZH2 had the shortest overall survival (*P* < 0.001, Supplementary Fig. S1c) and disease-free survival (*P* < 0.001, Fig. 4H) compared with patients with low TRIM25 or low EZH2 levels. Taken together, these observations suggest that TRIM25 regulates EZH2 levels in CRC cells by reducing the degradation of EZH2.

### TRIM25 blocks TRAF6-mediated ubiquitination of EZH2

**Fig. 5:**
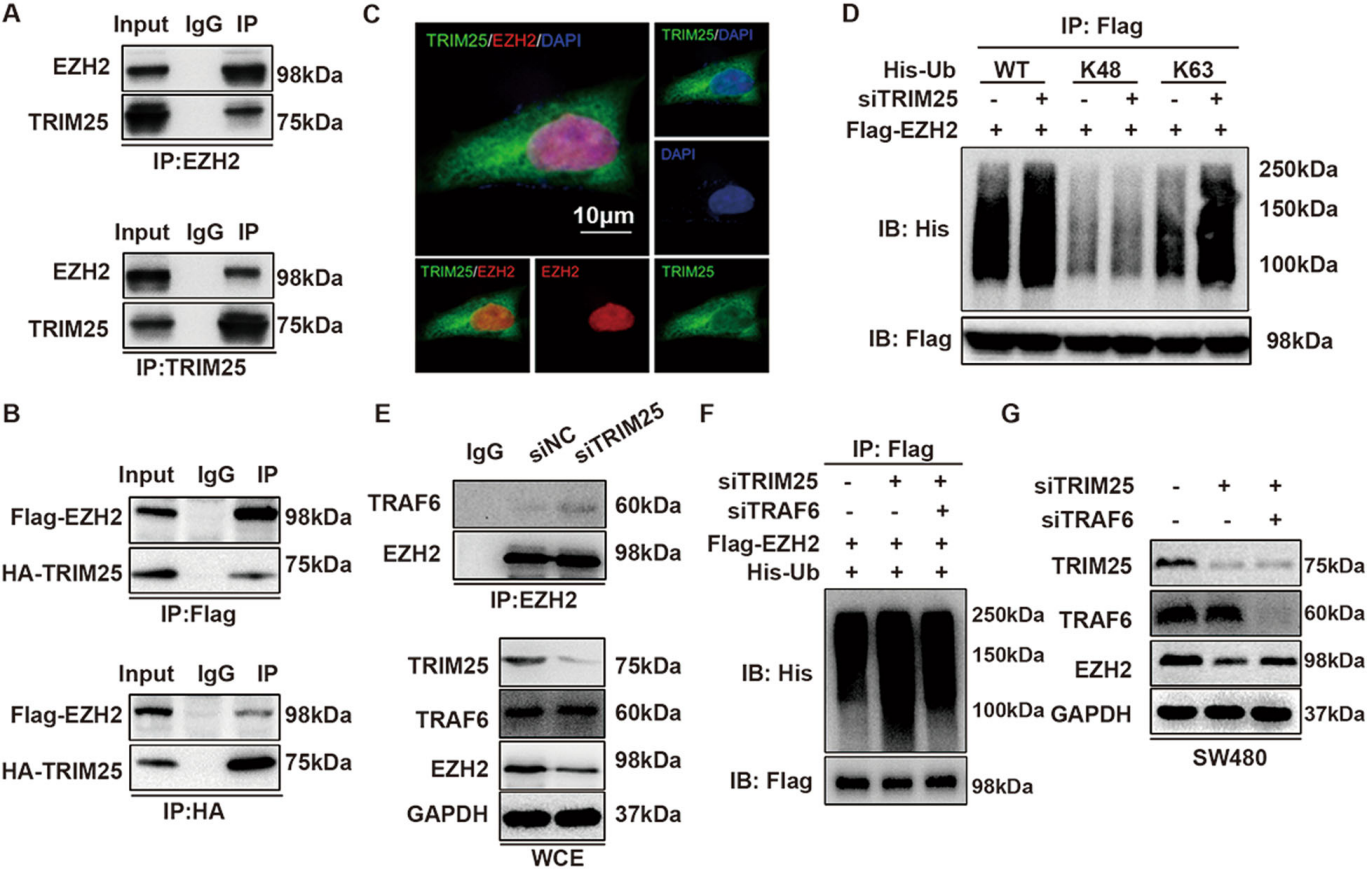
TRIM25 blocks TRAF6-mediated ubiquitination of EZH2. **A** Endogenous interaction between TRIM25 and EZH2 was determined using co-immunoprecipitation with anti-TRIM25 or anti-EZH2 antibodies in SW480 cells. **B** Exogenous interaction between TRIM25 and EZH2 was determined using co-immunoprecipitation with anti-Flag or anti-HA antibodies in HEK293T cells co-transfected with Flag-EZH2 and HA-TRIM25. **C** Immunofluorescence images showing colocalization of endogenous EZH2 and TRIM25 in SW480 cells. **D** Flag-EZH2, siTRIM25, His-tagged ubiquitin wild-type (WT) or mutation plasmids (K48 or K63 mutants) were transfected into HEK293T cells as the indicated combinations. Immunoprecipitation with anti-Flag antibodies was performed to detect the ubiquitination level of EZH2. **E** Immunoprecipitation assay using IgG and anti-EZH2 antibodies in SW480 cells transfected with siNC or siTRIM25, followed by western blotting analysis with the indicated antibodies. WCE: Whole-cell extracts. **F** Flag-EZH2, siTRIM25, siTRAF6, His-tagged ubiquitin wild-type (WT) were transfected into HEK293T cells as the indicated combinations. Immunoprecipitation with anti-Flag antibodies and western blotting with anti-His antibodies were performed to detect the ubiquitination level of EZH2 under different conditions. **G** Western blotting analysis of EZH2 in SW480 cells transfected with siTRIM25 and/or siTRAF6.

TRIM25 is an E3 ligase, therefore, we wondered if TRIM25 modulates EZH2 stability through the ubiquitin–proteasome pathway. First, we analyzed the interaction of TRIM25 with EZH2 in CRC cells. Using co-IP and immunofluorescence double staining, we verified the interaction between TRIM25 and EZH2 at both the exogenous and endogenous protein levels (Fig. 5A–C). Then, we performed in vivo ubiquitination assays in HEK293T cells transfected with siTRIM25, Flag-EZH2, His-tagged ubiquitin wild-type (WT), or mutation plasmids (K48 or K63 mutants). As shown in Fig. 5d, knockdown of TRIM25 in HEK293T cells increased the polyubiquitination of EZH2, indicating that EZH2 is not a substrate of TRIM25 E3 ligase. Interestingly, the enhanced EZH2 polyubiquitination by knockdown of TRIM25 was mainly extended through the K63-linkage instead of the K48-linkage. These findings were further validated in SW48 and SW480 cells (Supplementary Fig. S3a).

TNF receptor-associated factor 6 (TRAF6) is a member of the TNF receptor-associated factor (TRAF) protein family, and functions as an E3 ubiquitin ligase and a scaffold protein. TRAF6 mediates the K63-linked ubiquitination of EZH2 in prostate cancer^27^. Thus, we speculated whether TRIM25 is involved in TRAF6-mediated ubiquitination of EZH2. First, we confirmed the colocalization of endogenous EZH2 and TRAF6 in the nuclei of SW480 cells (Supplementary Fig. S3b). Then, we transfected siNC or siTRIM25 into SW480 cells. Western blotting analysis of the whole-cell extracts showed that knockdown of TRIM25 had no significant effect on TRAF6 but decreased the level of EZH2 in SW480 cells, which was consistent with the results shown in Fig. 4a. A further IP assay showed that TRAF6 protein could be detected in EZH2 immunoprecipitate and knockdown of TRIM25 enhanced the interaction between EZH2 and TRAF6, implying that TRIM25 might interfere with TRAF6-mediated EZH2 ubiquitination (Fig. 5e). To confirm the role of TRIM25 in TRAF6-mediated EZH2 ubiquitination, we blocked TRAF6 in TRIM25-knockdown SW480 cells. Co-IP and immunoblotting analysis showed that knockdown of TRIM25-induced EZH2 ubiquitination and degradation could be rescued using siTRAF6 (Fig. 5f, g). Taken together, these results suggest that TRIM25 stabilizes EZH2 by preventing TRAF6 binding to EZH2.

### EZH2 is required for TRIM25-induced OXA resistance in CRC both in vitro and in vivo

**Fig. 6:**
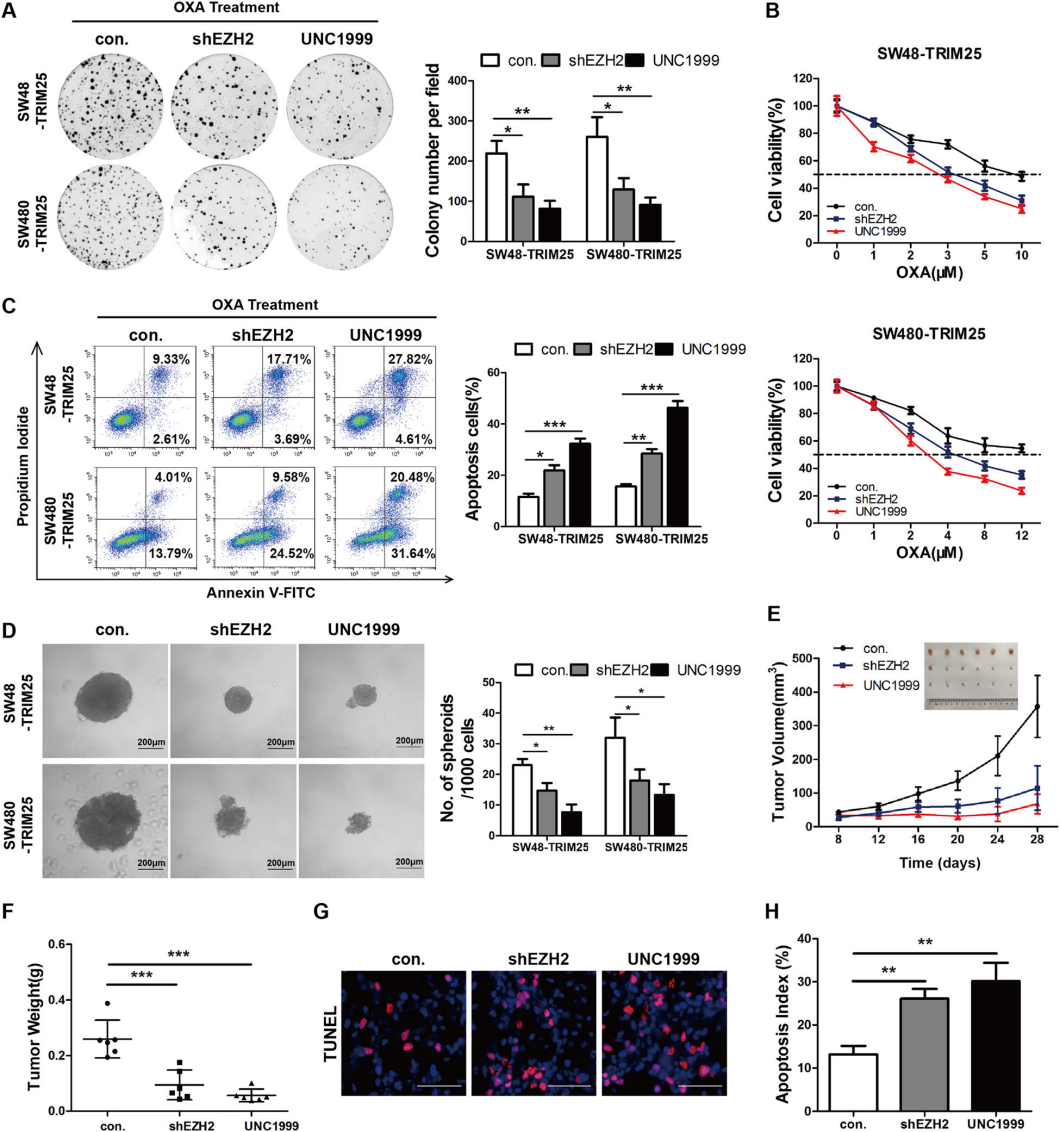
EZH2 is required for TRIM25-induced OXA resistance in CRC. **A** Colony-formation ability of the indicated cells treated with OXA (2 μM) for 14 days. Left panel: representative images; right panel: quantification of colony number. **B** CCK8 assay showing the IC50 values for OXA in the indicated cells treated with different concentrations of OXA or a combination with UNC1999 (1 μM) for 48 h. **C** Annexin V-FITC and PI staining showing apoptosis in the indicated CRC cells treated with OXA (30 μM) or a combination with UNC1999 (5 μM) for 48 h. Left panel: representative images; right panel: Quantification of apoptotic cells. **D** Representative images (left panel) and quantification (right panel) of the in vitro sphere formation assay using the indicated CRC cells. Scale bar, 200 μm. **E** Growth curves and tumor volume of xenograft tumors derived from TRIM25-overexpressing SW480 cells treated with control (PBS), shEZH2, or UNC1999 (*n* = 6 mice for each group). All mice were intraperitoneally injected with OXA (5 mg/kg, twice a week) one week after CRC cells inoculation. **F** Tumor weight of xenografts in each group. **G** Apoptotic cells in paraffin-embedded tumor sections derived from each group of SW480 cells-bearing mice were visualized using TUNEL staining. **H** Quantification of TUNEL-positive cells in the indicated group of tumor sections (*n* = 6). Data are represented as the mean ±SD. *P < 0.05, **P < 0.01, ***P < 0.001 for indicated comparison.

To further assess whether TRIM25 mediates OXA resistance via EZH2, we blocked EZH2 in TRIM25-overexpressing cells using shRNA or an EZH2 inhibitor (UNC1999). Colony formation, CCK8, and annexin V/PI apoptosis assays showed that inhibition of EZH2 significantly rescued the effect of TRIM25 overexpression on both the growth and antiapoptotic capacity of CRC cells treated with OXA (Fig. 6a–c). Besides, the sphere formation ability of SW48 and SW480 cells overexpressing TRIM25 was markedly suppressed by EZH2 inhibition (Fig. 6d).

Moreover, a nude mouse xenograft model was used to evaluate the effect of EZH2 inhibition on TRIM25-induced OXA resistance in vivo. Consistent with the in vitro findings, inhibition of EZH2 using shEZH2 or UNC1999 resulted in a significant reduction in tumor volume and weight when treated with OXA (Fig. 6E, F). Further TUNEL staining showed that inhibition of EZH2 resulted in more OXA-induced apoptosis compared with that in TRIM25-overexpressing cells in vivo, as demonstrated by a higher proportion of TUNEL positively stained cells after OXA treatment (Fig. 6G, H). Overall, EZH2 is essential for TRIM25-induced OXA resistance in CRC, and inhibition of EZH2 is expected to overcome TRIM25-induced OXA resistance in clinical practice.

## Discussion

**Fig. 7:**
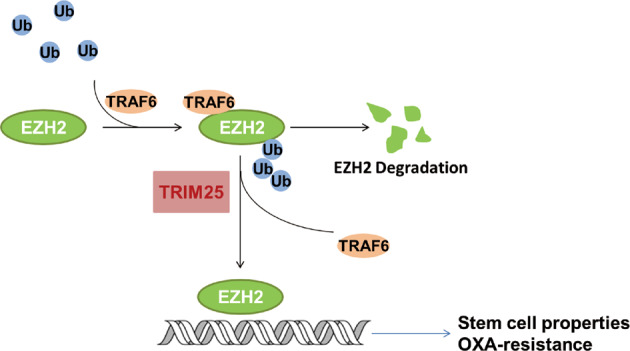
The working model depicting the role of TRIM25 in CRC cells. TRIM25 stabilizes EZH2 by blocking TRAF6-mediated ubiquitination for EZH2 degradation, and sustains stem cell properties to promote the resistance of CRC to OXA.

OXA is one of the most common chemotherapeutic agents used to treat CRC. However, resistance to OXA remains a major barrier to satisfactory tumor regression in patients with CRC. In the present study, we demonstrated that TRIM25 was highly expressed in CRC tissues, and high expression of TRIM25 was associated significantly with OXA resistance in patients with CRC. Mechanistically, we clarified that TRIM25 upregulates EZH2 levels by reducing TRAF6-mediated EZH2 ubiquitination and degradation, thus promoting the stem cell properties of CRC cells (Fig. 7). Our study revealed an important mechanism of OXA resistance and provided a promising strategy for CRC treatment.

TRIM25 is a member of the TRIM protein family, which represents the largest class of RING-containing E3 ubiquitin-ligases, and is involved in diverse cellular processes^28^. It was originally identified as an estrogen-responsive gene that is highly expressed in the breast, ovary, and uterus, and regulates various proteins involved in oncogenic signaling pathways. For example, in liver cancer, TRIM25 activates the Nrf2 signaling pathway and promotes tumor progression by interacting with and reducing the protein levels of Keap1^26^. Ken-ichi et al. reported that TRIM25 enhanced prostate cancer cell growth and cell survival by modulating p53 signals via interaction with G3BP2^28^. In the present study, we revealed that TRIM25 was a significant predictor of poor prognosis in patients with CRC treated with OXA, and high expression of TRIM25 predicted tumor recurrence, suggesting that TRIM25 is involved in OXA resistance. We then confirmed the important role of TRIM25 in sustaining CSC properties and inducing resistance of CRC cells to OXA-based treatment both in vitro and in vivo, and revealed a novel molecular mechanism based on the upregulation of EZH2 levels. These findings suggest that TRIM25 might be a potential therapeutic target for improving the response to OXA in patients with CRC.

Previous studies have identified the driving role of EZH2 in stem cell self-renewal. In CRC, EZH2 functions as a transcriptional repressor that downregulates IHH, a key gene responsible for normal colonocyte differentiation, resulting in an improved self-renewal capacity of CSCs^15^. In breast cancer, EZH2 increases NOTCH1 expression by directly binding to the NOTCH1 promoter and further promotes CSC properties or expands CSCs^17^. In addition to its canonical function via regulation of H3K27me3, EZH2 is reported to increase the self-renewal capacity of CSCs by binding to certain non-histone targets, such as STAT3, NF-κB, and β-catenin^29–31^. Therefore, targeting EZH2 is thought to be a rational and innovative strategy to treat CRC. In our study, IHC and immunofluorescence staining showed that TRIM25 is present in both the cytoplasmic and nuclear regions of CRC cells, suggesting that TRIM25 functions in the cytoplasm and/or nuclei of CRC cells. Subsequently, we found that EZH2 was a major target of TRIM25 in CRC, in which TRIM25 upregulated the protein level of EZH2. However, knockdown or overexpression of TRIM25 had no influence on the mRNA expression of EZH2, suggesting that TRIM25 regulates EZH2 in the posttranslational level. Posttranslational modifications, such as phosphorylation and glycosylation, are thought to be the main factors affecting the stability and activity of EZH2. For example, phosphorylation at Serine 21 mediated by AKT suppresses the activity of EZH2, thereby decreasing H3K27me3 levels^32^. Glycosylation of EZH2 is required for its stability and enzymatic activity^33,34^. Our study demonstrated the physical interaction and colocalization of EZH2 and TRIM25 in CRC cells, supporting the notion that TRIM25 might affect the function of EZH2 in CRC cells. We further confirmed that TRIM25 inhibited EZH2 ubiquitination, leading to increased stability of EZH2. Taken together, these results indicated that EZH2 is not degraded by TRIM25 as an E3 ligase, and suggest a new function of TRIM25 as an inhibitor of ubiquitination.

The regulation of EZH2 ubiquitination is complex, and several E3 ligases, such as Smurf2, β-TrCP, FOXP3, and Praja1, have been reported to be involved in these processes^35–38^. TRAF6 is an adaptor protein and E3 ubiquitin ligase that belongs to the TRAF family, and has a vital role in various solid tumors by activating multiple signaling pathways^39,40^. A recent study showed that TRAF6 could catalyze K63-linked polyubiquitination of EZH2 and promote its proteasome-dependent degradation in prostate cancer^27^. Consistent with the results reported by Lu et al., we confirmed the colocalization of endogenous EZH2 and TRAF6 in the nuclei of SW480 cells, suggesting that TRAF6 might modulate EZH2 function in the same way in CRC cells. As shown in Fig. 5e–f, knockdown of TRAF6 reversed the elevated ubiquitination and repression of EZH2 mediated by TRIM25 silencing, implying that TRIM25 regulates EZH2 via TRAF6. This regulation could be explained by two possible mechanisms. One possibility is that TRIM25 directly inhibits the catalytic activity of TRAF6. The E3 ubiquitin ligase activity of TRAF6 requires autoubiquitination through Lys63 (K63)-linked ubiquitin chains, which depend on its ring finger domain. Indeed, Lee et al.^41^ reported that deletion of TRIM25 inhibits TRAF6 ubiquitination and TRAF6-mediated NK-κB activation. The second possible mechanism is that TRIM25 might compete with TRAF6 to bind EZH2, which is supported by the data in the present study, showing that knockdown of TRIM25 promoted the interaction between TRAF6 and EZH2. These two mechanisms are not mutually exclusive, as suppression of TRAF6 activity might inhibit the binding of EZH2 to TRAF6. This is consistent with the previous reports that TRIM proteins can bind a protein to prevent its ubiquitination by another E3 ubiquitin ligase. For example, TRIM17 binds to BCL2A1 and prevents TRIM28-mediated ubiquitination and degradation of BCL2A1 in melanoma cells^42^. Besides, TRIM24 inhibits the degradation of dysbindin in cardiomyocytes in a similar manner^43^.

In our cohort, patients with CRC with higher TRIM25 levels showed a worse response to OXA treatment and poor prognosis. Thus, TRIM25 could be a valuable biomarker to identify patients who could benefit from OXA treatment. In addition, our findings provide new insights into the chemoresistance of CRC cells. The future development of inhibitors against TRIM25 might be an excellent strategy for CRC management in clinical practice.

In summary, this study demonstrated that TRIM25 maintained the stem cell properties and promoted the resistance of CRC cells to OXA by inhibiting EZH2 ubiquitination via TRAF6. Our findings identified TRIM25-TRAF6-EZH2 as a novel pathway of CRC chemoresistance, highlighting the prospect of blocking this pathway to improve the prognosis of patients with CRC. Importantly, clinical evidence suggested a correlation between TRIM25 levels and the response of patients with CRC to OXA treatment. Our study contributes to a better understanding of OXA resistance and indicates that TRIM25 might be a new target for CRC treatment in the future.

## Supplementary information

Supplementary Figures

Supplementary Tables
