# Supplementary material for: TRIM25 regulates oxaliplatin resistance in colorectal cancer by promoting EZH2 stability
Source: Cell Death Dis. 2021 May 8;12(5):463. doi: 10.1038/s41419-021-03734-4 (PMC8106682; doi:10.1038/s41419-021-03734-4)
Supplement: Supplementary file 1 — Supplementary Figures [file 41419_2021_3734_MOESM1_ESM.docx]

**Supplementary Figure S1**. TRIM25 predicts recurrence and poor prognosis of patients with CRC treated with OXA-based chemotherapy.


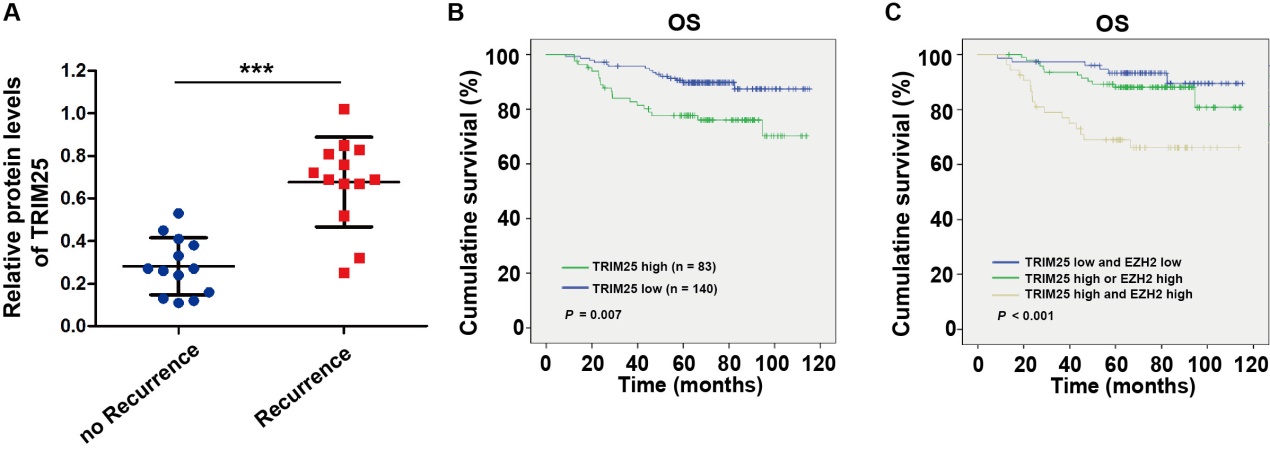


**Supplementary Figure S2**. Western blotting analysis of stem cell markers in TRIM25-knockdown /-overexpressing CRC cells.
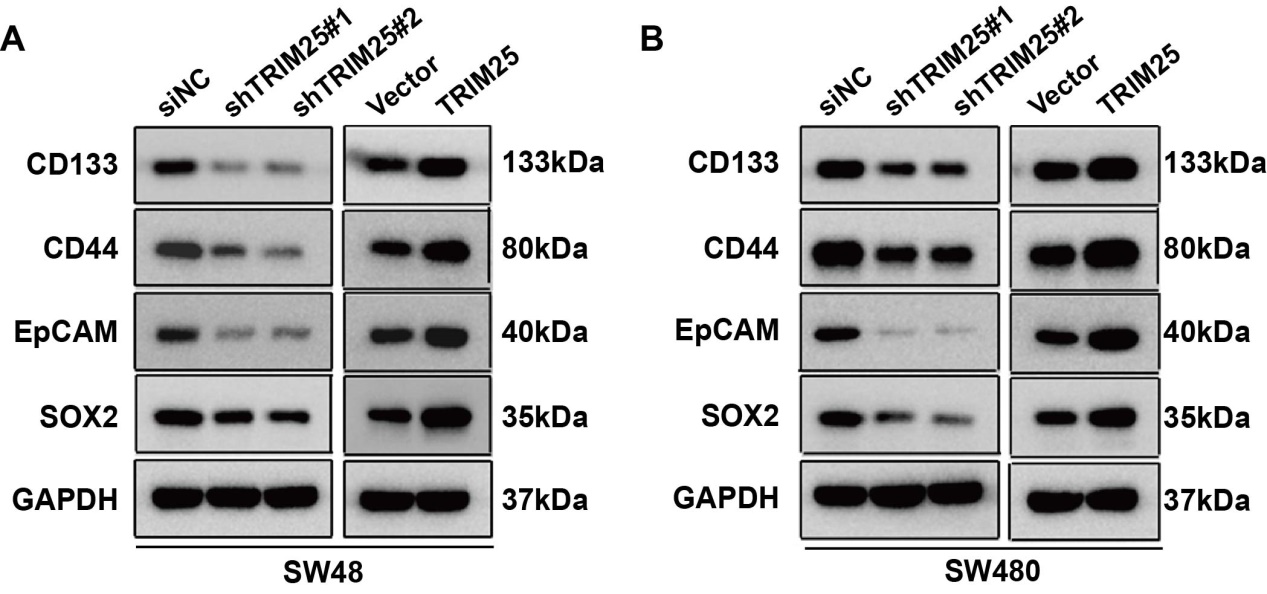


**Supplementary Figure S3**. TRIM25 regulates the ubiquitination of EZH2.

**
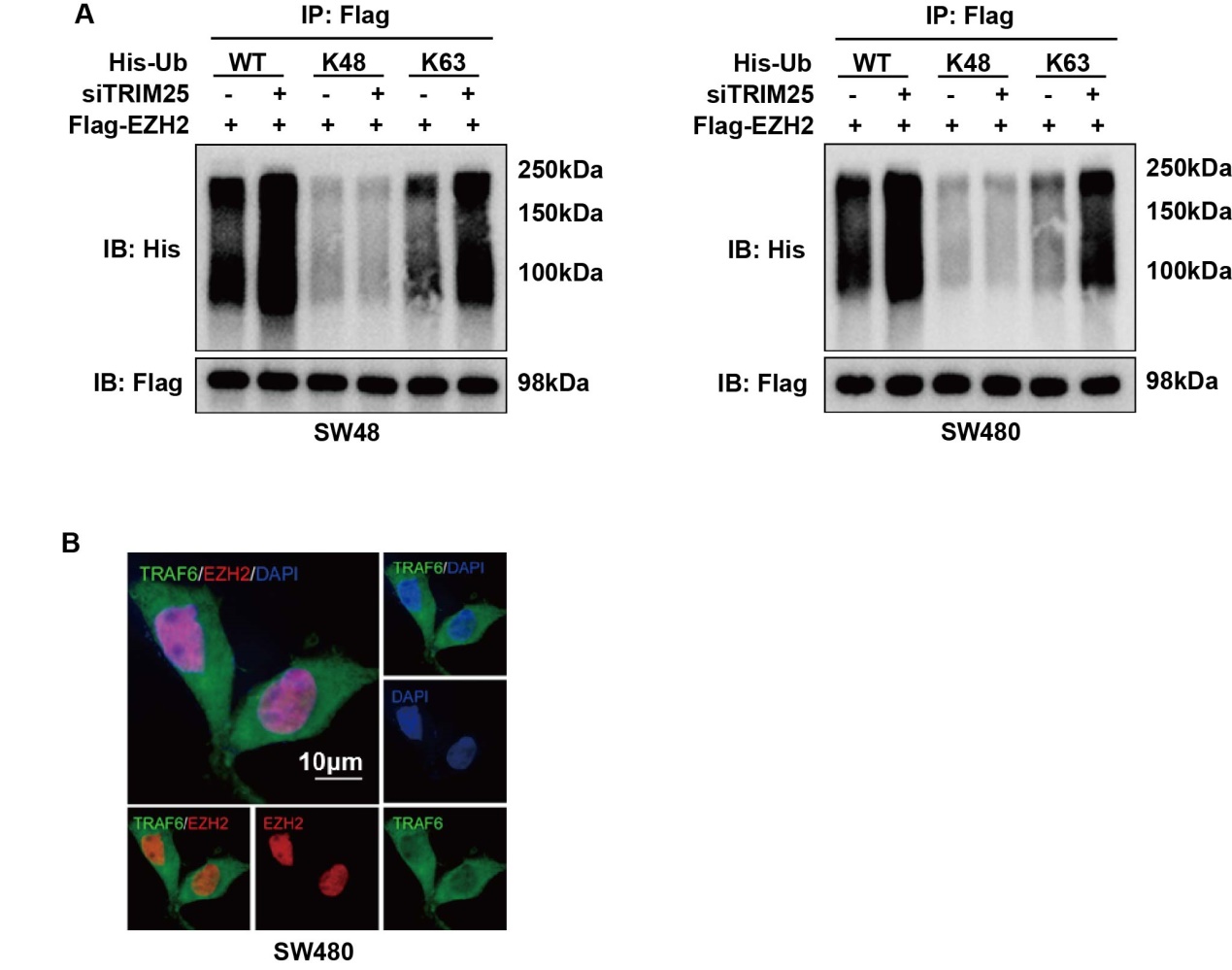
**
