# Supplementary material for: TRIM25 regulates oxaliplatin resistance in colorectal cancer by promoting EZH2 stability
Source: Cell Death Dis. 2021 May 8;12(5):463. doi: 10.1038/s41419-021-03734-4 (PMC8106682; doi:10.1038/s41419-021-03734-4)
Supplement: Supplementary file 2 — Supplementary Tables [file 41419_2021_3734_MOESM2_ESM.docx]

**Supplementary Table 1.** Patient characteristics.

| Characteristic | Total (n = 223), % |
| --- | --- |
| Age (years) |  |
| <56 | 108 (48.4) |
| ≥56 | 115 (51.6) |
| Sex |  |
| Male | 125 (56.1) |
| Female | 98 (43.9) |
| Tumor location |  |
| Right-sided | 87 (39.0) |
| Left-sided | 136 (61.0) |
| Primary tumor size |  |
| <4.5 cm | 117 (52.5) |
| ≥4.5 cm | 106 (47.5) |
| T stage |  |
| T1-2 | 91 (40.8) |
| T3-4 | 132 (59.2) |
| N stage |  |
| N1 | 150 (67.3) |
| N2 | 73 (32.7) |
| TNM stage |  |
| T1-3N1M0 | 65 (29.1) |
| T4NanyM0 or TanyN2M0 | 158 (70.9) |

**Supplementary Table 2.** Univariate and multivariate analyses for disease-free survival.

| Factors | Univariate | |  | Multivariate | |
| --- | --- | --- | --- | --- | --- |
|  | 5-year DFS (%) | *P*-value |  | HR (95% CI) | *P*-value |
|  |  |  |  |  |  |
| Age (years) |  | 0.041 |  | 1.708 (0.944-3.091) | 0.077 |
| ˂56 | 84.2 |  |  |  |  |
| ≥56 | 75 |  |  |  |  |
| Sex |  | 0.068 |  | 1.659(0.908-3.032) | 0.100 |
| Male | 74.9 |  |  |  |  |
| Female | 85.4 |  |  |  |  |
| Tumor location |  | 0.616 |  |  |  |
| Right-side | 76.6 |  |  |  |  |
| Left-side | 81.4 |  |  |  |  |
| Tumor size |  | 0.952 |  |  |  |
| <4.5cm | 79.1 |  |  |  |  |
| ≥4.5cm | 80.1 |  |  |  |  |
| TNM stage |  | 0.055 |  | 1.785(0.828-3.850) | 0.139 |
| T1-3N1M0 | 89.2 |  |  |  |  |
| T4NanyM0 or TanyN2M0 | 75.7 |  |  |  |  |
| TRIM25 expression |  | 0.006 |  | 1.927 (1.083-3.428) | 0.026 |
| High | 84.1 |  |  |  |  |
| Low | 71.8 |  |  |  |  |

**Supplementary Table 3.** Target sequences for shRNAs used in this study.

| Target shRNA sequence | |
| --- | --- |
| shTRIM25#1 | GGGUGGGCGUGCUUCUCAACU |
| shTRIM25#2 | GGGAUGAGUUCGAGUUUCUGG |
| shEZH2 | CCCAACATAGATGGACCAAAT |
